# Supplementary material for: Care-seeking for diarrheal illness: A systematic review and meta-analysis
Source: PLOS Glob Public Health. 2025 Apr 22;5(4):e0004483. doi: 10.1371/journal.pgph.0004483 (PMC12013865; doi:10.1371/journal.pgph.0004483)
Supplement: S1 Checklist — (PDF) [file pgph.0004483.s001.pdf]

PRISMA 2020 Checklist

Section and Topic	Item #	Checklist item	Location where item is reported
**TITLE**			
Title	1	Identify the report as a systematic review.	**Title**
**ABSTRACT**			
Abstract	2	See the PRISMA 2020 for Abstracts checklist.	**Abstract**
**INTRODUCTION**			
Rationale	3	Describe the rationale for the review in the context of existing knowledge.	**Introduction**, paragraphs 1-3
Objectives	4	Provide an explicit statement of the objective(s) or question(s) the review addresses.	**Introduction**, paragraph 4
**METHODS**			
Eligibility criteria	5	Specify the inclusion and exclusion criteria for the review and how studies were grouped for the syntheses.	**Methods**, section "Systematic review", paragraph 2
Information sources	6	Specify all databases, registers, websites, organisations, reference lists and other sources searched or consulted to identify studies. Specify the date when each source was last searched or consulted.	**Supplementary methods**, section "Systematic review search methods"
Search strategy	7	Present the full search strategies for all databases, registers and websites, including any filters and limits used.	**Supplementary methods**, section "Systematic review search methods"
Selection process	8	Specify the methods used to decide whether a study met the inclusion criteria of the review, including how many reviewers screened each record and each report retrieved, whether they worked independently, and if applicable, details of automation tools used in the process.	**Methods**, section "Systematic review", paragraph 3
Data collection process	9	Specify the methods used to collect data from reports, including how many reviewers collected data from each report, whether they worked independently, any processes for obtaining or confirming data from study investigators, and if applicable, details of automation tools used in the process.	**Methods**, section "Systematic review", paragraph 3
Data items	10a	List and define all outcomes for which data were sought. Specify whether all results that were compatible with each outcome domain in each study were sought (e.g. for all measures, time points, analyses), and if not, the methods used to decide which results to collect.	**Methods**, section "Systematic review", paragraph 3

PRISMA 2020 Checklist

Section and Topic	Item #	Checklist item	Location where item is reported
			and **S1 data**
	10b	List and define all other variables for which data were sought (e.g. participant and intervention characteristics, funding sources). Describe any assumptions made about any missing or unclear information.	**Methods**, section "Systematic review", paragraph 3 and **S1 Data**
Study risk of bias assessment	11	Specify the methods used to assess risk of bias in the included studies, including details of the tool(s) used, how many reviewers assessed each study and whether they worked independently, and if applicable, details of automation tools used in the process.	**Methods,** section "Systematic review", paragraph 4
Effect measures	12	Specify for each outcome the effect measure(s) (e.g. risk ratio, mean difference) used in the synthesis or presentation of results.	Not applicable
Synthesis methods	13a	Describe the processes used to decide which studies were eligible for each synthesis (e.g. tabulating the study intervention characteristics and comparing against the planned groups for each synthesis (item #5)).	**Methods**, section "Data cleaning", paragraph 1
	13b	Describe any methods required to prepare the data for presentation or synthesis, such as handling of missing summary statistics, or data conversions.	**Methods**, section "Systematic review", paragraph 2; **Methods**, section "Data cleaning", paragraphs 3-4
	13c	Describe any methods used to tabulate or visually display results of individual studies and syntheses.	Not applicable
	13d	Describe any methods used to synthesize results and provide a rationale for the choice(s). If meta-analysis was performed, describe the model(s), method(s) to identify the presence and extent of statistical heterogeneity, and software package(s) used.	**Methods** section, "Meta-analysis", paragraph 2
	13e	Describe any methods used to explore possible causes of heterogeneity among study results (e.g. subgroup analysis, meta-regression).	**Methods** section, "Meta-analysis", paragraph 2
	13f	Describe any sensitivity analyses conducted to assess robustness of the synthesized results.	**Methods** section, "Meta-analysis", paragraph 2
Reporting bias assessment	14	Describe any methods used to assess risk of bias due to missing results in a synthesis (arising from reporting biases).	Not applicable (missing data were generally

Section and Topic	Item #	Checklist item	Location where item is reported
			not reported by the included studies)
Certainty assessment	15	Describe any methods used to assess certainty (or confidence) in the body of evidence for an outcome.	We did not conduct a certainty assessment
**RESULTS**			
Study selection	16a	Describe the results of the search and selection process, from the number of records identified in the search to the number of studies included in the review, ideally using a flow diagram.	**Results**, paragraph 1 and Figure 1
	16b	Cite studies that might appear to meet the inclusion criteria, but which were excluded, and explain why they were excluded.	**Results**, paragraph 1, **S1 Data**
Study characteristics	17	Cite each included study and present its characteristics.	**Results**, paragraph 1, **S1 Data,** Table 1
Risk of bias in studies	18	Present assessments of risk of bias for each included study.	**Results**, section "Trends in care seeking at hospitals or clinics in the unadjusted data", paragraph 3
Results of individual studies	19	For all outcomes, present, for each study: (a) summary statistics for each group (where appropriate) and (b) an effect estimate and its precision (e.g. confidence/credible interval), ideally using structured tables or plots.	**S1 Appendix,** Figures S9-S14
Results of syntheses	20a	For each synthesis, briefly summarise the characteristics and risk of bias among contributing studies.	**Results,** section "Trends in care seeking at hospitals or clinics in the unadjusted data", paragraph 3<br>**S1 Appendix**, Figure S5
	20b	Present results of all statistical syntheses conducted. If meta-analysis was done, present for each the summary estimate and its precision (e.g. confidence/credible interval) and measures of statistical heterogeneity. If comparing groups, describe the direction of the effect.	**Results**, section "Adjusted estimates of care seeking at hospitals or

Section and Topic	Item #	Checklist item	Location where item is reported
			clinics", paragraphs 1-2, **S1 Appendix,** Figures S9-S14
	20c	Present results of all investigations of possible causes of heterogeneity among study results.	**Results**, section "Factors associated with variation in care seeking at hospitals or clinics in LMICs", paragraphs 1-3, Tables 2-3, **S1 Appendix** Tables S2-S4
	20d	Present results of all sensitivity analyses conducted to assess the robustness of the synthesized results.	Not applicable
Reporting biases	21	Present assessments of risk of bias due to missing results (arising from reporting biases) for each synthesis assessed.	Not applicable
Certainty of evidence	22	Present assessments of certainty (or confidence) in the body of evidence for each outcome assessed.	A formal certainty assessment was not conducted. "Confidence" is represented by 95% Confidence Interval (95% CI) throughout the abstract, results, figures, and tables.
**DISCUSSION**			
Discussion	23a	Provide a general interpretation of the results in the context of other evidence.	**Discussion**, paragraphs 1-3
	23b	Discuss any limitations of the evidence included in the review.	**Discussion**, paragraphs 3-4
	23c	Discuss any limitations of the review processes used.	**Discussion**, paragraph 4
	23d	Discuss implications of the results for practice, policy, and future research.	**Discussion**, paragraphs 5-7
**OTHER INFORMATION**			

PRISMA 2020 Checklist

Section and Topic	Item #	Checklist item	Location where item is reported
Registration and protocol	24a	Provide registration information for the review, including register name and registration number, or state that the review was not registered.	**Methods**, section "Systematic review", paragraph 1
	24b	Indicate where the review protocol can be accessed, or state that a protocol was not prepared.	**Methods**, section "Systematic review", paragraph 1
	24c	Describe and explain any amendments to information provided at registration or in the protocol.	Not applicable
Support	25	Describe sources of financial or non-financial support for the review, and the role of the funders or sponsors in the review.	**Funding** section
Competing interests	26	Declare any competing interests of review authors.	**Conflicts of Interest** section
Availability of data, code and other materials	27	Report which of the following are publicly available and where they can be found: template data collection forms; data extracted from included studies; data used for all analyses; analytic code; any other materials used in the review.	**Methods**, section "Data and code availability", **S1 Data**

From: Page MJ, McKenzie JE, Bossuyt PM, Boutron I, Hoffmann TC, Mulrow CD, et al. The PRISMA 2020 statement: an updated guideline for reporting systematic reviews. BMJ 2021;372:n71. doi: 10.1136/bmj.n71
For more information, visit: http://www.prisma-statement.org/
