# Supplementary material for: Care-seeking for diarrheal illness: A systematic review and meta-analysis
Source: PLOS Glob Public Health. 2025 Apr 22;5(4):e0004483. doi: 10.1371/journal.pgph.0004483 (PMC12013865; doi:10.1371/journal.pgph.0004483)
Supplement: S1 Appendix — Supplementary methods, including systematic review search terms, and supplementary figures and tables. (PDF) [file pgph.0004483.s002.pdf]

Care seeking for diarrheal illness: a systematic review and meta-analysis

S1 Appendix

Table of Contents

Section A: Supplementary Methods **2**

Systematic review search methods 2

Section B: Supplementary Figures **5**

Figure A. Data coverage by geography 5

Figure B. Data coverage over time 6

Figure C. Proportion that sought care at a hospital or clinic by region 7

Figure D. Proportion that sought care at a hospital or clinic by income group 8

Figure E. Proportion that sought care by study quality in LMICs 9

Figure F. Proportion that sought care by source and income group in LMICs 10

Figure G. Proportion that sought care by source and case definition in LMICs 11

Figure H. Proportion that sought care by source for clinic-based surveillance studies 12

Figure I. Forest plot of care seeking at a hospital or clinic in LMICs 13

Figure J. Forest plot of hypothetical care seeking at a hospital or clinic in LMICs 16

Figure K. Forest plot of hypothetical care seeking at a hospital or clinic in HICs 17

Figure L. Forest plot of care seeking for general diarrhea in LMICs 18

Figure M. Forest plot of care seeking for severe diarrhea or cholera in LMICs 21

Figure N. Forest plot of care seeking for gastroenteritis or other etiologies in LMICs 21

Section C: Supplementary Tables **22**

Table A. Standardized categories for sources of care and examples from studies 22

Table B. Univariate analyses of factors associated with variation in care seeking 23

Table C. Tests for potential confounding with severe diarrhea or cholera case definition 24

Table D. Sub-analyses of associations with outbreak context and recall period 25

Section A: Supplementary Methods

Systematic review search methods

Search methods for identification of studies:
To identify studies to include or consider for this systematic review, the review team worked with a medical librarian (AGS) to develop detailed search strategies for each database. The PRISMA-S extension was followed for search reporting. The medical librarian (AGS) developed the search for PubMed (NLM) and translated the search for every database searched. The PubMed (NLM) search strategy was reviewed by the research team to check for accuracy and term relevancy, and all final searches were peer-reviewed by another medical librarian following the PRESS checklist. The databases included in this search are PubMed (NLM), Embase (Elsevier, embase.com), Web of Science (Clarivate Analytics), and Global Index Medicus (World Health Organization), which were searched using a combination of keywords and subject headings. Searches were limited to articles published since 2000, in line with the review team's body of literature. All final searches were performed on January 27, 2023; searches were then updated on September 3, 2024. The full search strategies as reported by the librarian are provided below.

Search strings:

PubMed (NLM)

(diarrhea*[tiab] OR diarrhoea*[tiab] OR cholera[tiab] OR diarrhea[mesh] OR cholera[mesh]) AND (care seeking[tiab] OR healthcare seeking[tiab] OR seeking healthcare[tiab] OR seek* care[tiab] OR sought care[tiab] OR health seeking[tiab] OR health care utilization[tiab] OR health care utilisation[tiab] OR healthcare utilization[tiab] OR healthcare utilisation[tiab] OR health care use[tiab] OR healthcare use[tiab] OR health services utilization[tiab] OR health services utilisation[tiab] OR health services use[tiab] OR treatment seeking[tiab] OR seek* treatment[tiab] OR health behavior[tiab] OR health behaviour[tiab] OR "patient acceptance of health care"[mesh] OR "health services accessibility"[mesh] OR "health knowledge, attitudes, practice"[mesh] or "health behavior"[mesh:noexp]) AND (2000:2024[pdat])

Embase (Elsevier, embase.com)

#1	(diarrhea*:ab,ti or diarrhoea*:ab,ti or cholera:ab,ti or diarrhea/exp or cholera/exp)
#2	(‘care seeking’:ab,ti or ‘healthcare seeking’:ab,ti or (seeking NEAR/3 healthcare):ab,ti or (seek* NEAR/3 care):ab,ti or (sought NEAR/3 care):ab,ti or ‘health seeking’:ab,ti or ‘health care utilization’:ab,ti or ‘health care utilisation’:ab,ti or ‘healthcare utilization’:ab,ti or ‘healthcare utilisation’:ab,ti or ‘health care use’:ab,ti or ‘healthcare use’:ab,ti or ‘health services utilization’:ab,ti or ‘health services utilisation’:ab,ti or ‘health services use’:ab,ti or ‘treatment seeking’:ab,ti or (seek* NEAR/3 treatment):ab,ti or ‘health behavior’:ab,ti or ‘health behaviour’:ab,ti or ‘patient attitude’/de or ‘health care utilization’/exp or ‘help seeking behavior’/exp or ‘health behavior’/de)
#3	#1 and #2 and [2000-2024]/py

Web of Science (Clarivate Analytics)

#1	TS=(diarrhea* or diarrhoea* or cholera)
#2	TS=(“care seeking” or “healthcare seeking” or (seeking NEAR/3 healthcare) or (seek* NEAR/3 care) or (sought NEAR/3 care) or “health seeking” or “health care utilization” or “health care utilisation” or “healthcare utilization” or “healthcare utilisation" or “health care use” or “healthcare use” or “health services utilization” or “health services utilisation" or “health services use” or “treatment seeking” or (seek* NEAR/3 treatment) or “health behavior” or “health behaviour”)
#3	DOP=(2000/2024)
#4	#1 and #2 and #3

Global Index Medicus (WHO)

(tw:(diarrhea* or diarrhoea* or cholera)) AND (tw:("care seeking" or "healthcare seeking" or "seeking healthcare" or "seek care" or "seeking care" or "sought care" or "health seeking" or "health care utilization" or “health care utilisation” or “healthcare utilization” or “healthcare utilisation" or “health care use” or “healthcare use” or “health services utilization” or “health services utilisation" or “health services use” or “treatment seeking” or "seek treatment" or "seeking treatment")) AND (year_cluster:[2000 TO 2024])

Summary of the search results from databases:
PubMed (NLM, 1809-present): 2372 results
Embase (Elsevier, embase.com, 1974-present): 3561 results
Web of Science (Clarivate Analytics, 1900-present): 1123 results
Global Index Medicus (World Health Organization): 13 results

The search resulted in 7069 studies and 1881 duplicate studies were found and omitted using Covidence or by manual identification. This resulted in 5188 records to screen from databases or registers.

Section B: Supplementary Figures

Figure A. Data coverage by geography

Number of observations in the primary dataset at each administrative level as defined by GADM (https://gadm.org/) by country. Countries with >10 observations displayed as 10.

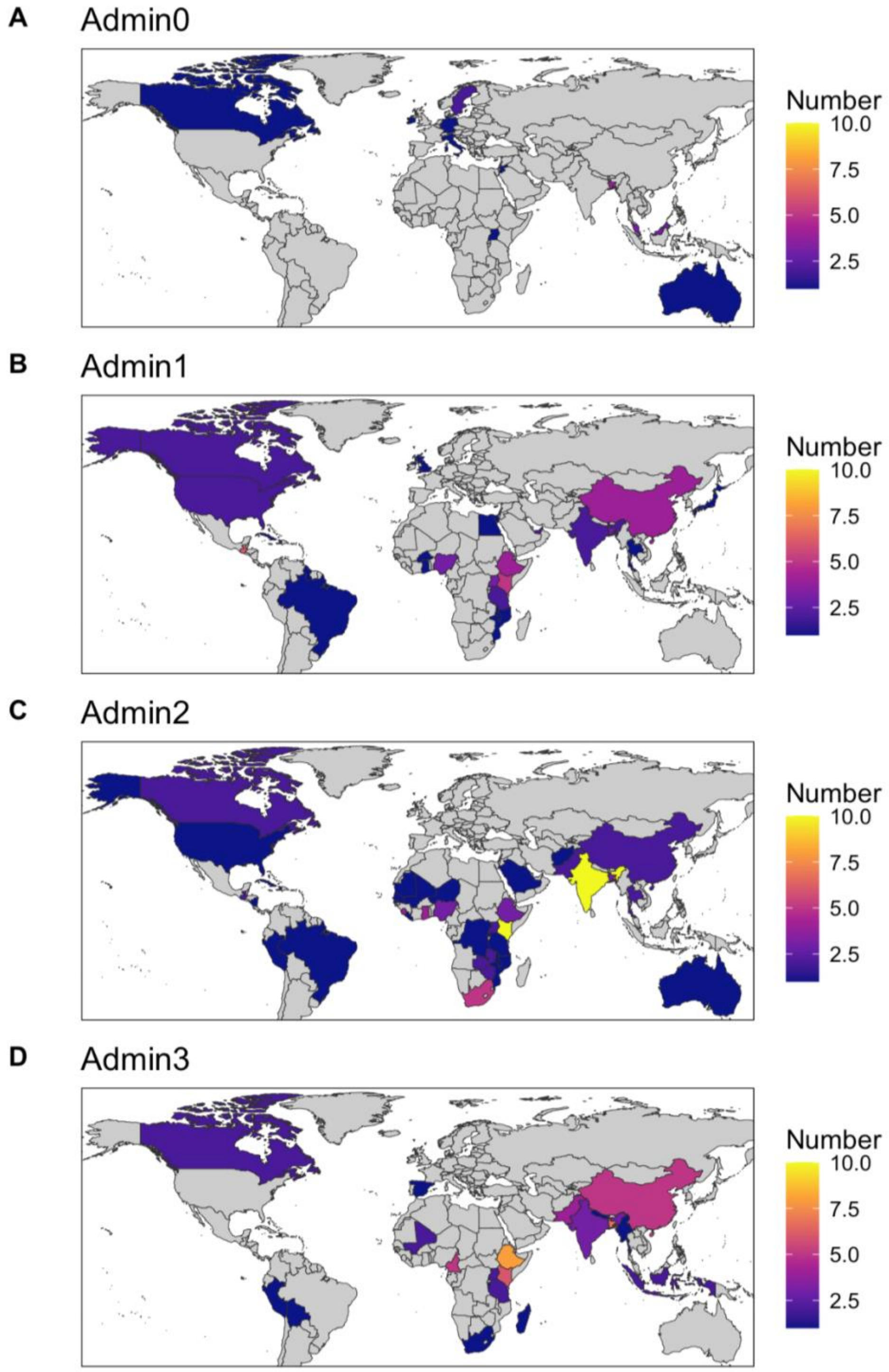

Figure B. Data coverage over time

Number of observations in the primary dataset at each administrative level as defined by GADM (https://gadm.org/) by country within different time periods. Countries with >10 observations displayed as 10. Year represents the year sampling was completed. Excludes 6 studies missing a study end date.

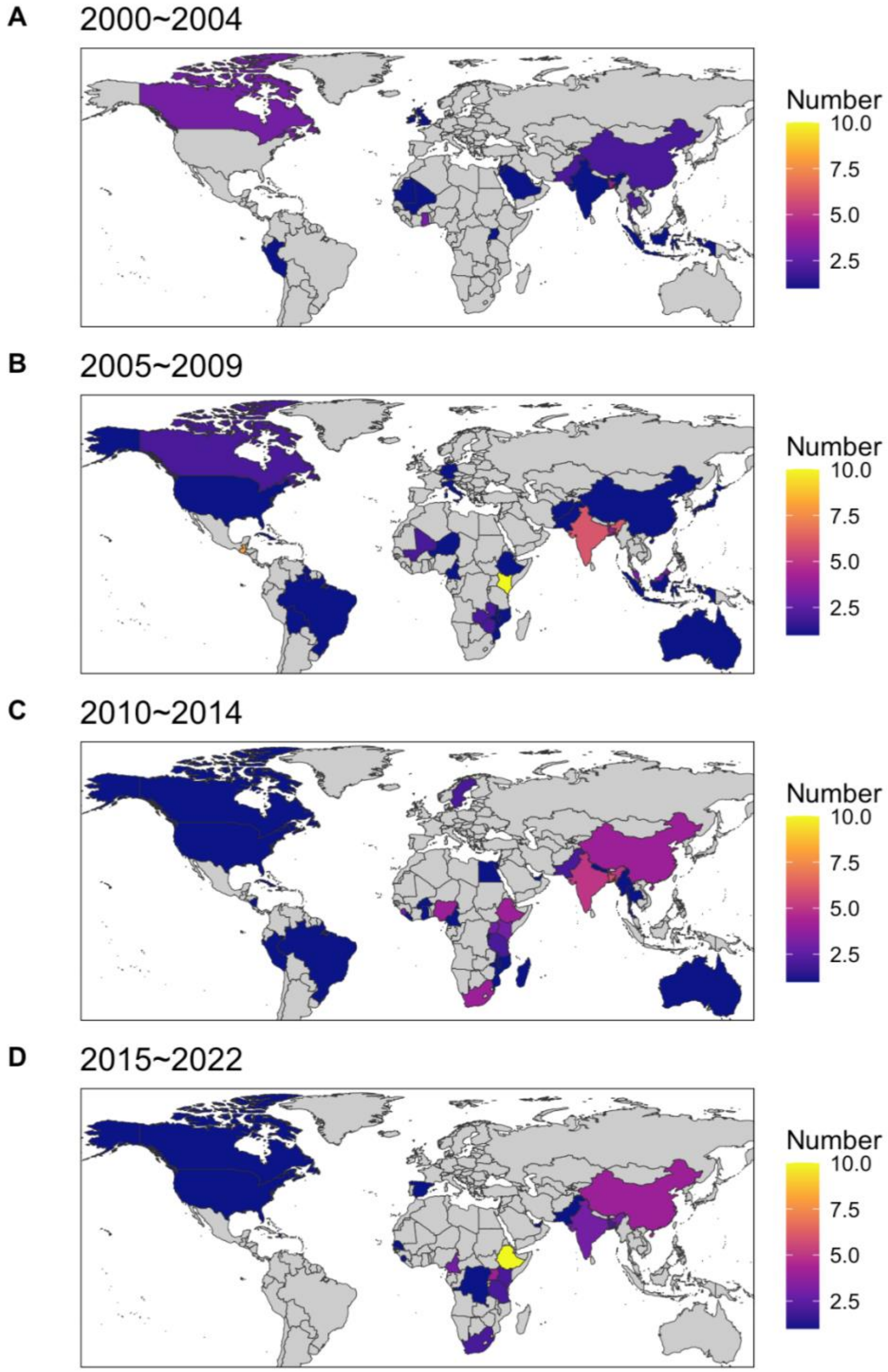

Figure C. Proportion that sought care at a hospital or clinic by region

Proportion of respondents that reported seeking care for diarrheal illness at a hospital or clinic grouped by A) geographic region and B) geographic region and case definition. Each point represents an observation. Boxes represent the median and interquartile range (IQR) of the proportion for each group. Lines extend from the top and bottom of box to the largest proportion value no further than 1.5 * IQR from the box.

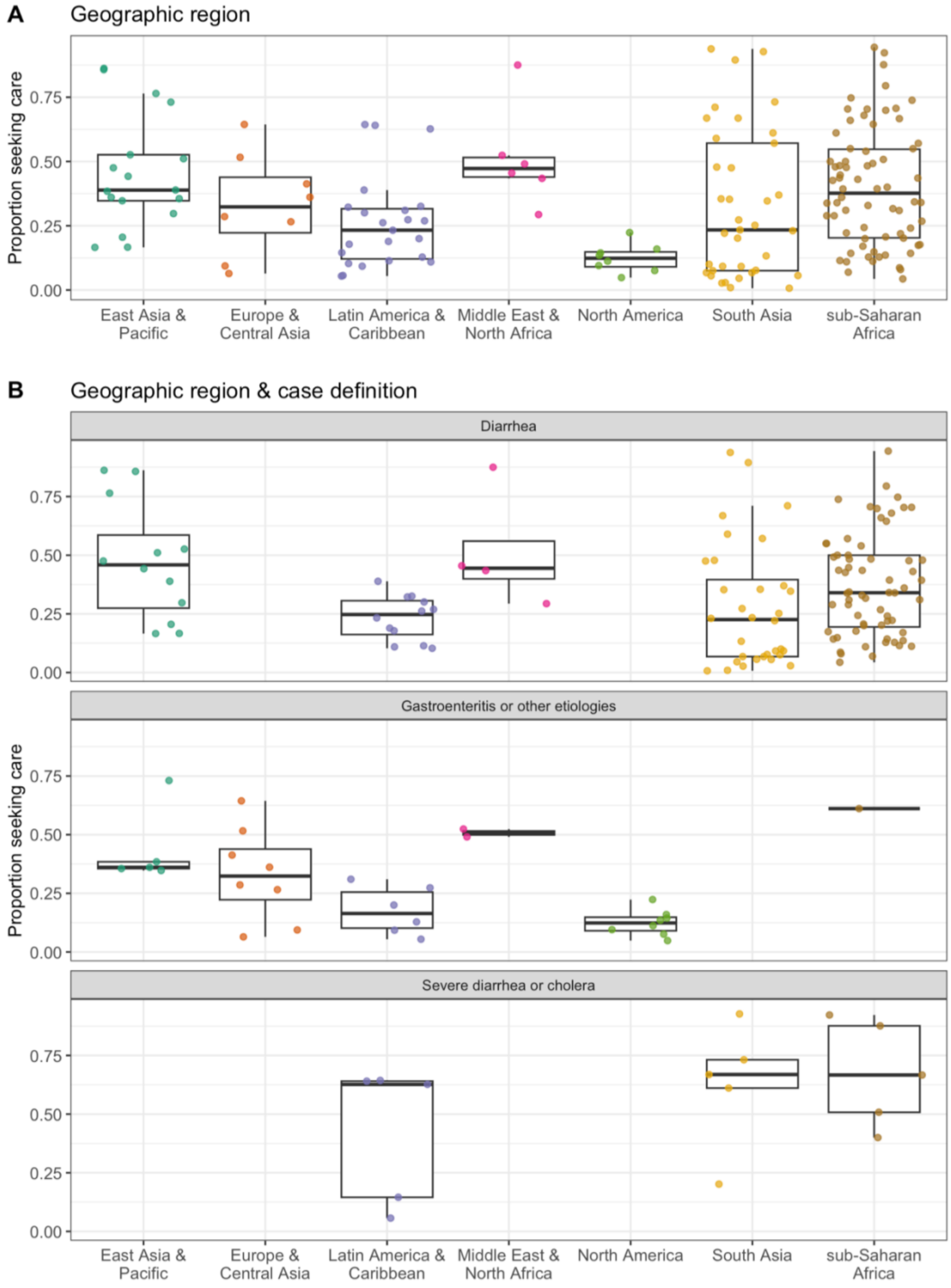

Figure D. Proportion that sought care at a hospital or clinic by income group

Proportion of respondents that reported seeking care for diarrheal illness at a hospital or clinic grouped by A) World Bank income group and B) World Bank 2023-2024 income group and case definition. Each point represents an observation. Boxes represent the median and interquartile range (IQR) of the proportion for each group. Lines extend from the top and bottom of box to the largest proportion value no further than 1.5 * IQR from the box.

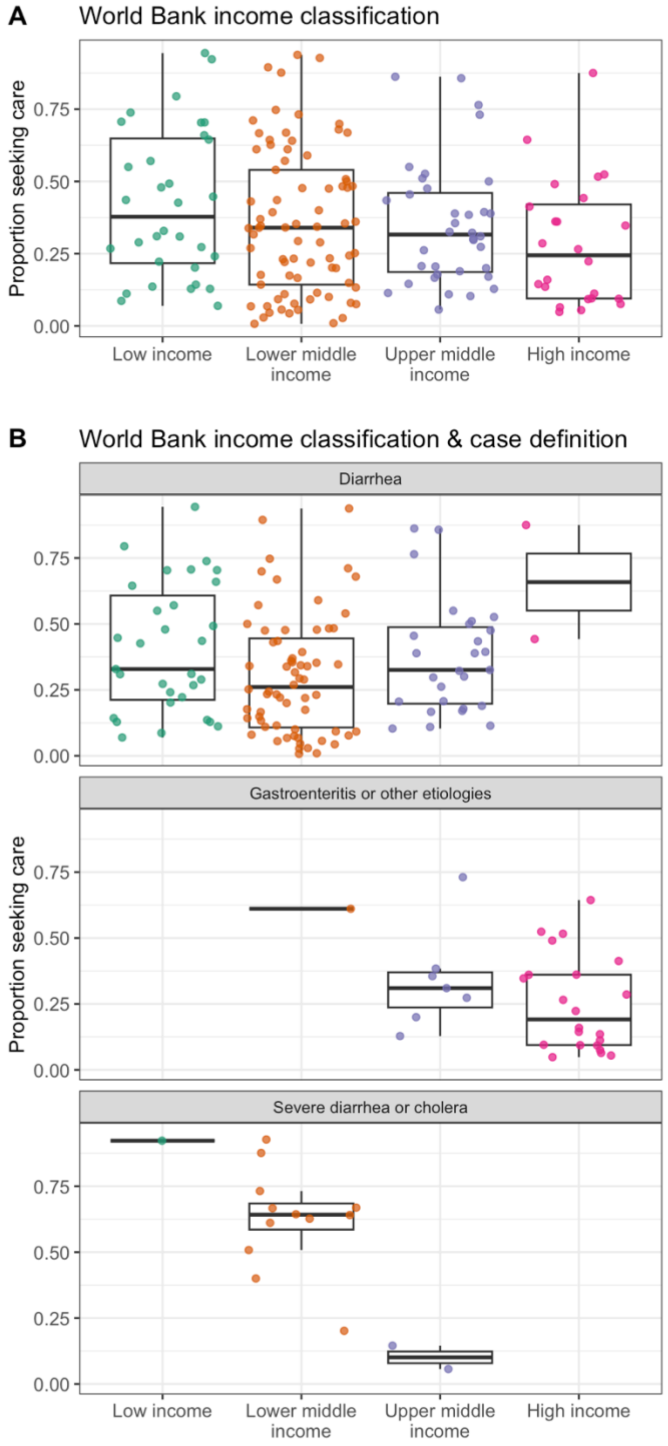

Figure E. Proportion that sought care by study quality in LMICs

Proportion of respondents in low- and middle-income countries (LMICs) that reported seeking care for diarrheal illness at a hospital or clinic grouped by A) overall quality score, B) whether the data is internally consistent (i.e., numbers for the proportion seeking care were identical throughout the abstract, text, and tables), C) whether the manuscript included a justification for their sample size, and D) whether the non-response rate was reported. Overall quality score is the sum of B-D. Each point represents an observation. Boxes represent the median and interquartile range (IQR) of the proportion for each group. Lines extend from the top and bottom of box to the largest proportion value no further than 1.5 * IQR from the box.

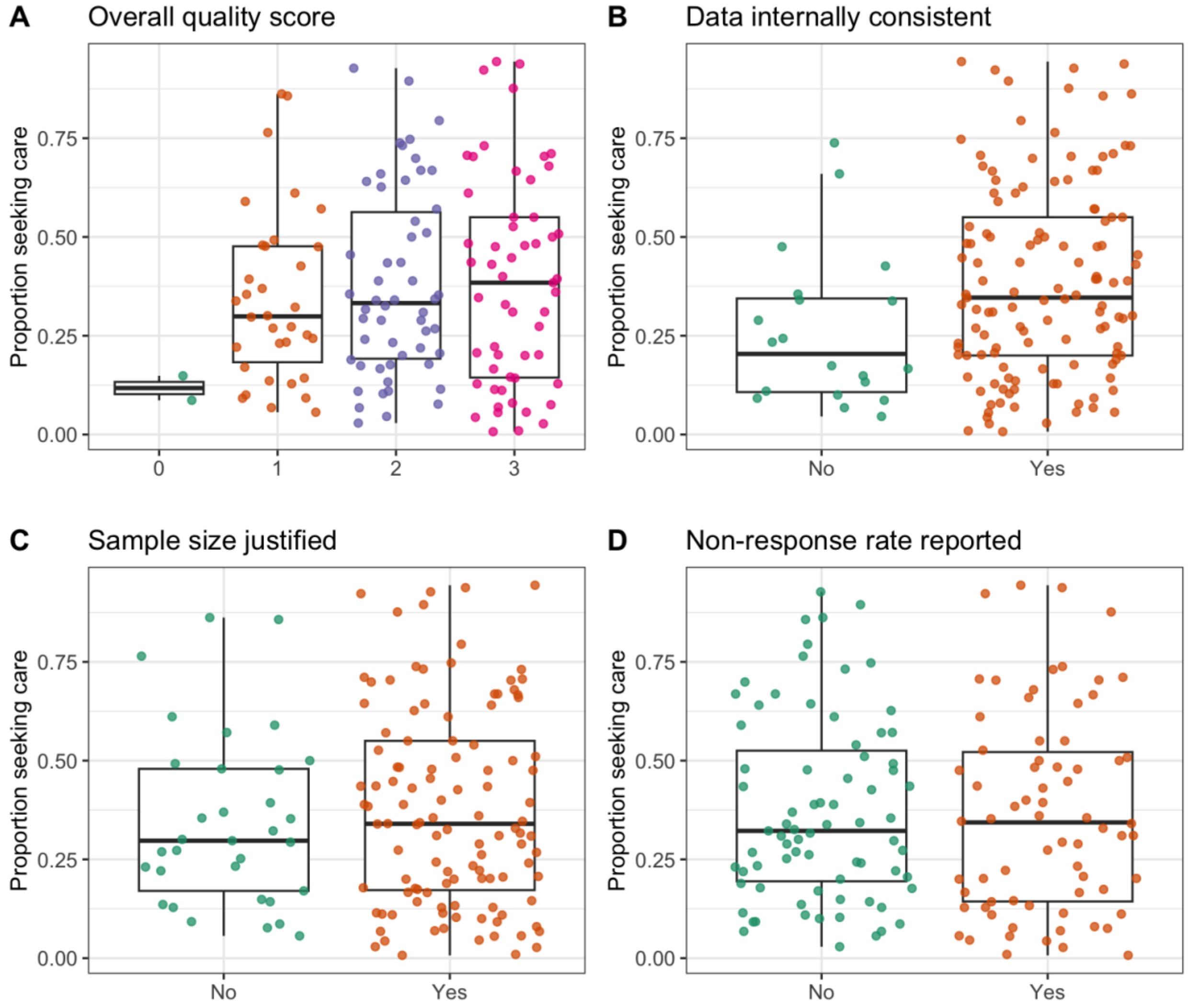

Figure F. Proportion that sought care by source and income group in LMICs

Proportion of respondents in LMICs that reported seeking care for diarrheal illness at all categories of care sources by the World Bank income group of the country the study was conducted in. Each point represents an observation. Boxes represent the median and interquartile range (IQR) of the proportion for each group. Lines extend from the top and bottom of box to the largest proportion value no further than 1.5 * IQR from the box.

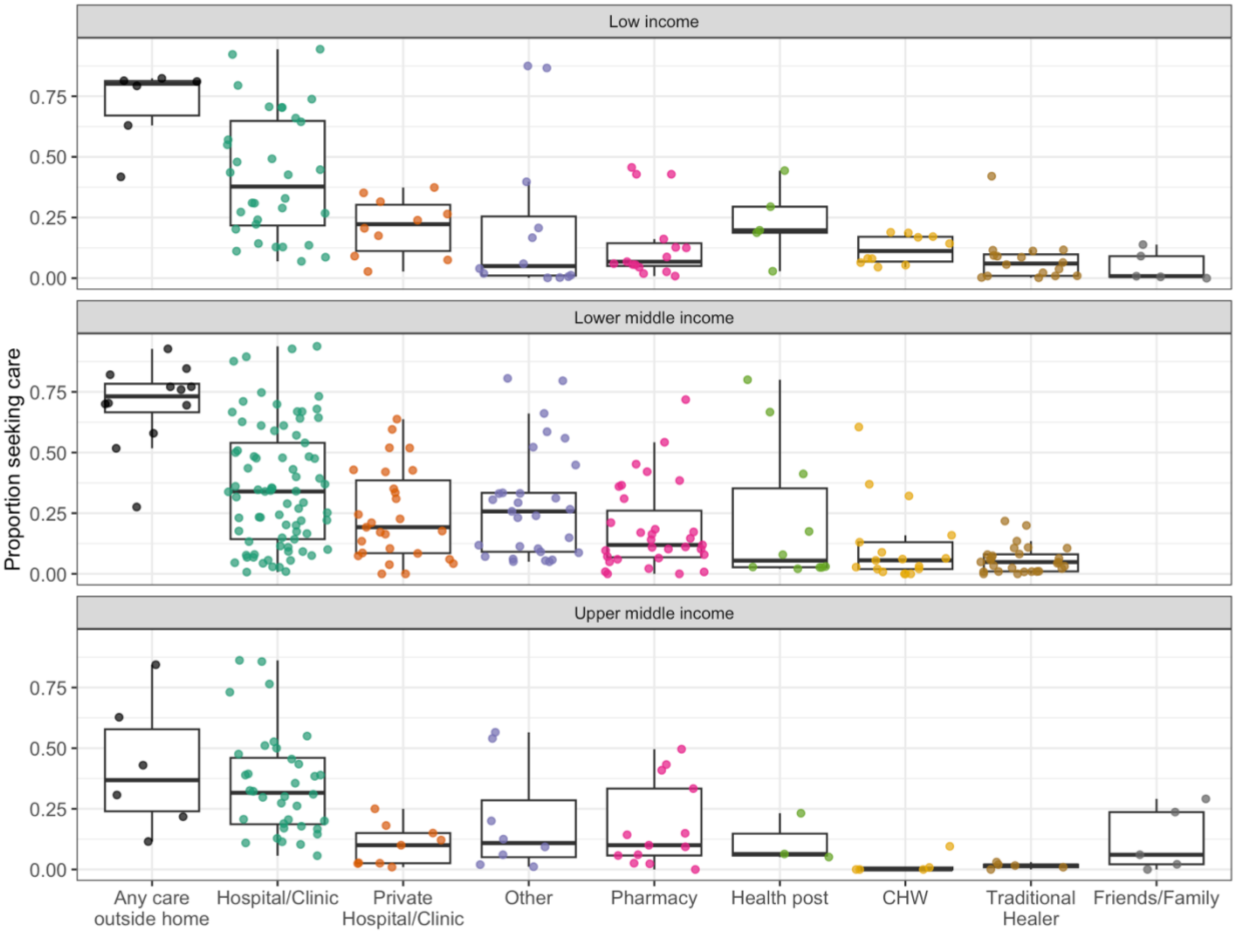

Figure G. Proportion that sought care by source and case definition in LMICs

Proportion of respondents in LMICs that reported seeking care for diarrheal illness at all categories of care sources by diarrhea case definition. Each point represents an observation. Boxes represent the median and interquartile range (IQR) of the proportion for each group. Lines extend from the top and bottom of box to the largest proportion value no further than 1.5 * IQR from the box.

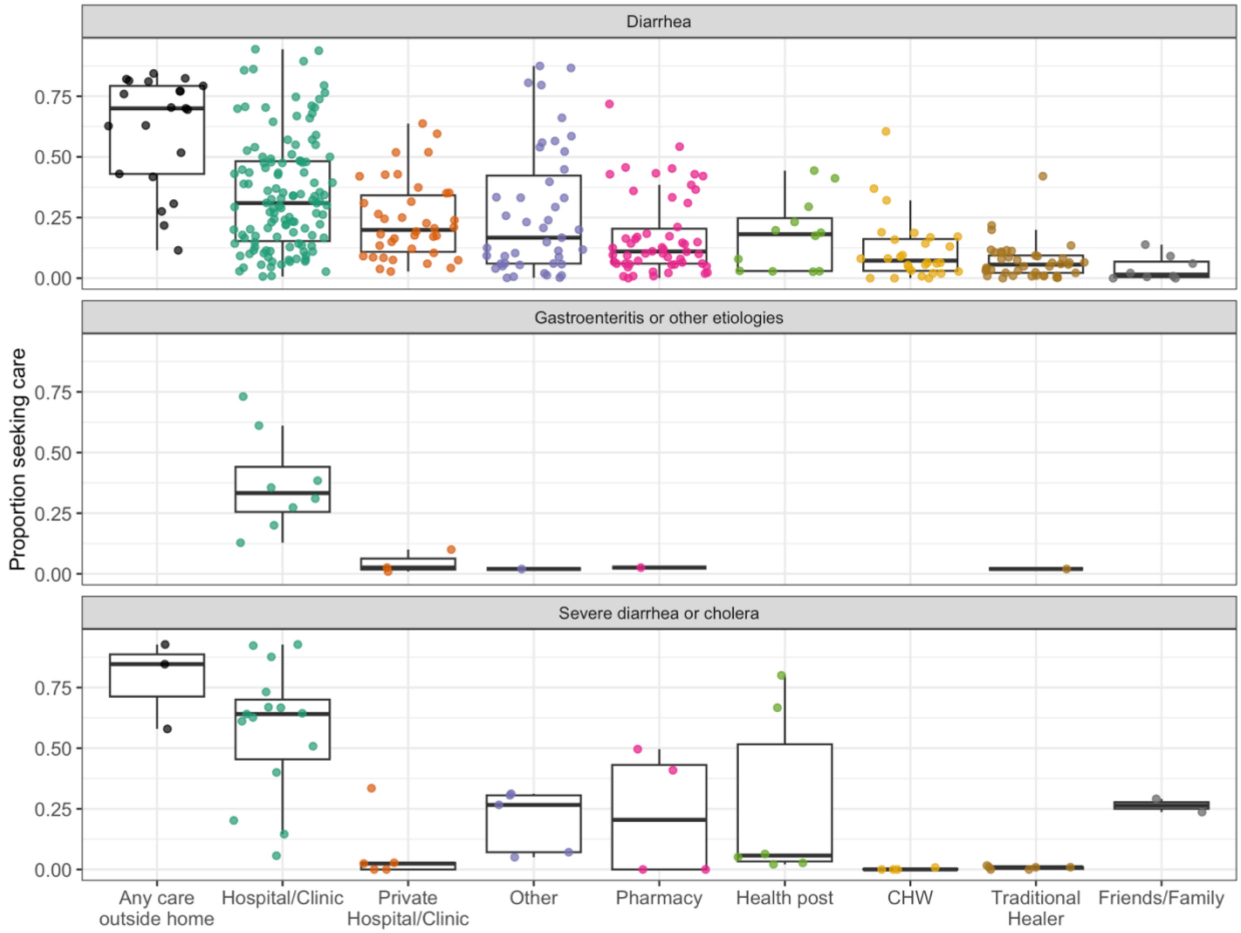

Figure H. Proportion that sought care by source for clinic-based surveillance studies

Proportion of respondents in clinic-based surveillance studies in LMICs that reported seeking care for diarrheal illness at all categories of care sources A) as their first source of care and B) prior to their current hospital or clinic visit. Each point represents an observation. Boxes represent the median and interquartile range (IQR) of the proportion for each group. Lines extend from the top and bottom of box to the largest proportion value no further than 1.5 * IQR from the box.

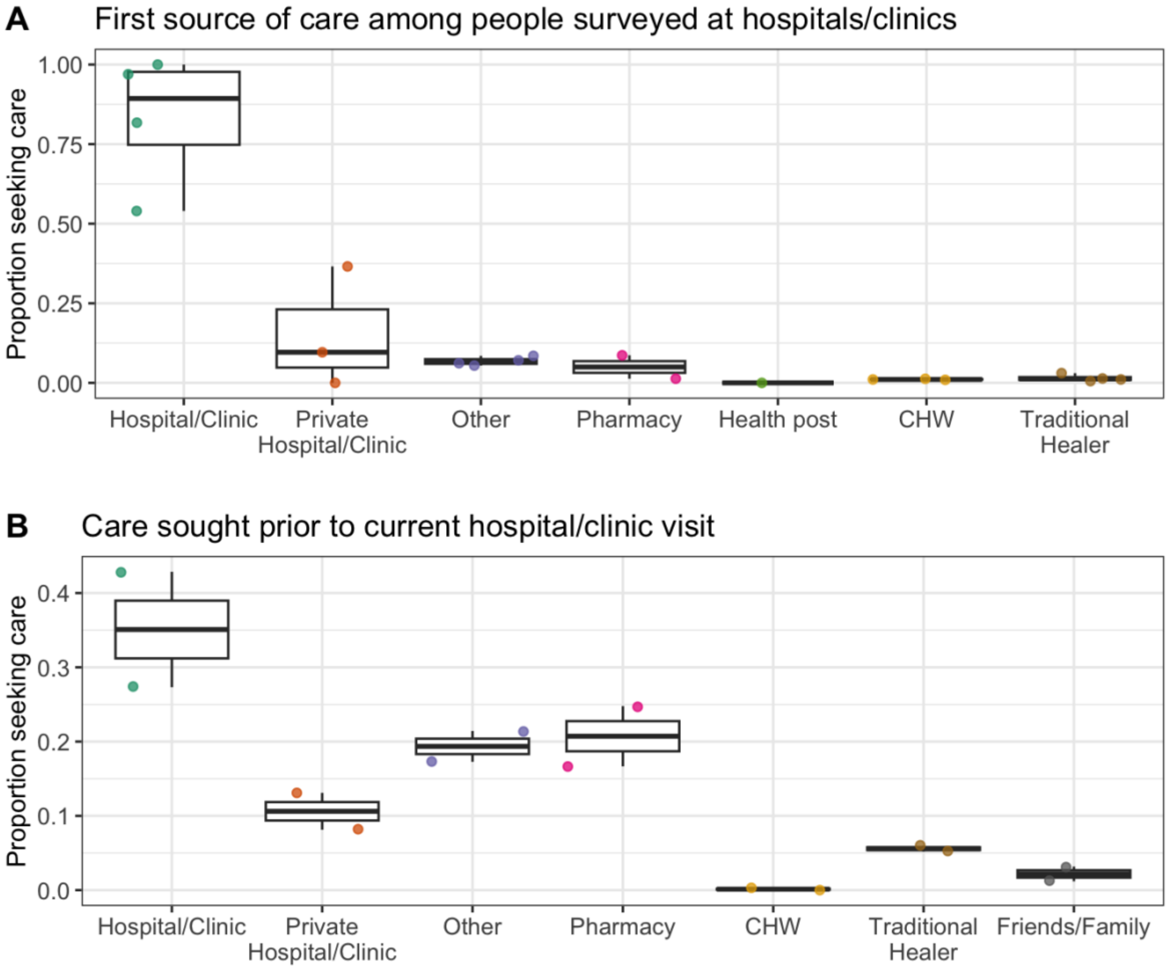

Figure I. Forest plot of care seeking at a hospital or clinic in LMICs

Estimated proportion that sought care for an actual episode of diarrheal illness at a hospital or clinic across the 145 observations included in the random-effects meta-analysis.

Study	Events	Total	Proportion	95%-CI
IND (125) Child, Diarrhea	58	8038	0.01	[0.01; 0.01]
BGD (26) Child, Diarrhea	1	106	0.01	[0.00; 0.05]
IND (134) Child, Diarrhea	41	1508	0.03	[0.02; 0.04]
IND (145) Combination, Diarrhea	2	69	0.03	[0.00; 0.10]
KEN (15) Combination, Diarrhea	5	115	0.04	[0.01; 0.10]
IND (85) Child, Diarrhea	98	2145	0.05	[0.04; 0.06]
BGD (31) Child, Diarrhea	15	270	0.06	[0.03; 0.09]
BGD (28) Combination, Diarrhea	291	5156	0.06	[0.05; 0.06]
GTM (14) Child, Severe diarrhea or cholera	21	371	0.06	[0.04; 0.09]
IND (24) Combination, Diarrhea	29	428	0.07	[0.05; 0.10]
BGD (92) Child, Diarrhea	275	4049	0.07	[0.06; 0.08]
UGA (178) Child, Diarrhea	49	706	0.07	[0.05; 0.09]
IND (100) Child, Diarrhea	211	2800	0.08	[0.07; 0.09]
IND (175) Self, Diarrhea	2	26	0.08	[0.01; 0.25]
KEN (15) Child, Diarrhea	15	188	0.08	[0.05; 0.13]
LBR (101) Child, Diarrhea	20	231	0.09	[0.05; 0.13]
PAK (163) Combination, Diarrhea	26	283	0.09	[0.06; 0.13]
BGD (116) Child, Diarrhea	62	671	0.09	[0.07; 0.12]
PAK (163) Child, Diarrhea	63	629	0.10	[0.08; 0.13]
GTM (17) Combination, Diarrhea	22	213	0.10	[0.07; 0.15]
GTM (17) Self, Diarrhea	40	366	0.11	[0.08; 0.15]
CMR (127) Child, Diarrhea	367	3342	0.11	[0.10; 0.12]
UGA (178) Child, Diarrhea	185	1657	0.11	[0.10; 0.13]
BRA (108) Combination, Diarrhea	61	535	0.11	[0.09; 0.14]
TZA (23) Child, Diarrhea	21	183	0.11	[0.07; 0.17]
LCA (64) Combination, Gastroenteritis or other etiologies	5	39	0.13	[0.04; 0.27]
MWI (36) Child, Diarrhea	136	1059	0.13	[0.11; 0.15]
MLI (171) Child, Diarrhea	65	506	0.13	[0.10; 0.16]
BGD (129) Child, Diarrhea	333	2504	0.13	[0.12; 0.15]
UGA (203) Child, Diarrhea	61	449	0.14	[0.11; 0.17]
KEN (123) Combination, Diarrhea	7	49	0.14	[0.06; 0.27]
UGA (203) Combination, Diarrhea	20	140	0.14	[0.09; 0.21]
GTM (14) Child, Severe diarrhea or cholera	16	110	0.15	[0.09; 0.23]
KEN (91) Child, Diarrhea	121	814	0.15	[0.12; 0.17]
MMR (32) Child, Diarrhea	42	253	0.17	[0.12; 0.22]
THA (93) Self, Diarrhea	1	6	0.17	[0.00; 0.64]
ZAF (192) Child, Diarrhea	55	323	0.17	[0.13; 0.22]
KEN (13) Child, Diarrhea	78	448	0.17	[0.14; 0.21]
ZMB (33) Child, Diarrhea	392	2220	0.18	[0.16; 0.19]
GTM (17) Self, Diarrhea	31	174	0.18	[0.12; 0.24]
GTM (17) Combination, Diarrhea	21	111	0.19	[0.12; 0.27]
GHA (12) Child, Diarrhea	22	110	0.20	[0.13; 0.29]
JAM (103) Combination, Gastroenteritis or other etiologies	10	50	0.20	[0.10; 0.34]
IND (176) Child, Severe diarrhea or cholera	47	233	0.20	[0.15; 0.26]
UGA (137) Child, Diarrhea	65	322	0.20	[0.16; 0.25]
CHN (102) Combination, Diarrhea	105	511	0.21	[0.17; 0.24]
ZAF (22) Combination, Diarrhea	18	87	0.21	[0.13; 0.31]
GHA (166) Combination, Diarrhea	16	73	0.22	[0.13; 0.33]
BGD (25) Child, Diarrhea	21	95	0.22	[0.14; 0.32]
MLI (171) Child, Diarrhea	28	126	0.22	[0.15; 0.30]
BGD (99) Self, Diarrhea	48	208	0.23	[0.18; 0.29]
NIC (9) Child, Diarrhea	54	232	0.23	[0.18; 0.29]
BGD (92) Child, Diarrhea	762	3258	0.23	[0.22; 0.25]
UGA (46) Child, Diarrhea	165	685	0.24	[0.21; 0.27]
KEN (131) Combination, Diarrhea	3339	13724	0.24	[0.24; 0.25]
PAK (192) Child, Diarrhea	809	3210	0.25	[0.24; 0.27]
GTM (17) Child, Diarrhea	44	168	0.26	[0.20; 0.34]
ETH (82) Child, Diarrhea	242	904	0.27	[0.24; 0.30]
HTI (141) Combination, Diarrhea	14	52	0.27	[0.16; 0.41]
AFG (72) Child, Diarrhea	30	110	0.27	[0.19; 0.37]

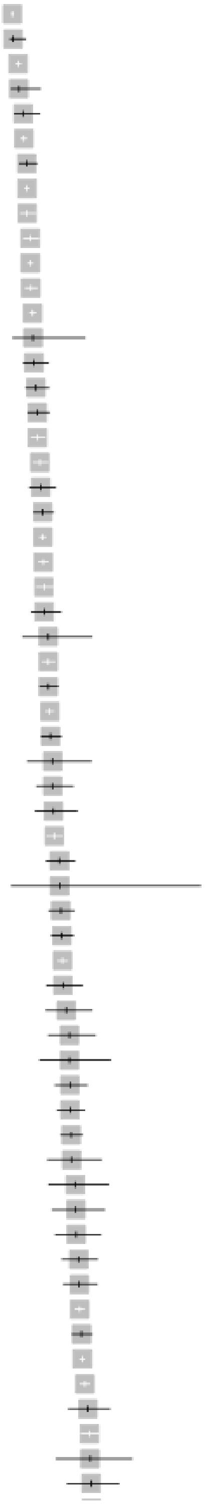

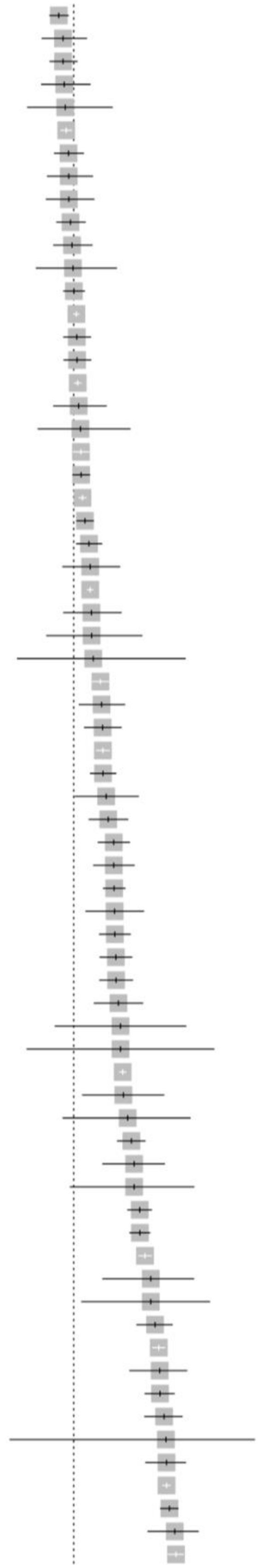

CUB (97) Combination, Gastroenteritis or other etiologies	186	680	0.27 [0.24; 0.31]
GHA (12) Child, Diarrhea	37	128	0.29 [0.21; 0.38]
MDG (96) Child, Diarrhea	96	332	0.29 [0.24; 0.34]
JOR (135) Combination, Diarrhea	32	109	0.29 [0.21; 0.39]
THA (167) Combination, Diarrhea	11	37	0.30 [0.16; 0.47]
PER (192) Child, Diarrhea	632	2102	0.30 [0.28; 0.32]
ETH (88) Child, Diarrhea	94	304	0.31 [0.26; 0.36]
GRD (118) Combination, Gastroenteritis or other etiologies	40	129	0.31 [0.23; 0.40]
ETH (3) Child, Diarrhea	36	116	0.31 [0.23; 0.40]
ZMB (89) Child, Diarrhea	101	319	0.32 [0.27; 0.37]
BRA (192) Child, Diarrhea	58	180	0.32 [0.25; 0.40]
PER (39) Child, Diarrhea	14	43	0.33 [0.19; 0.49]
ETH (168) Child, Diarrhea	196	596	0.33 [0.29; 0.37]
KEN (131) Child, Diarrhea	2981	8817	0.34 [0.33; 0.35]
KEN (113) Child, Diarrhea	126	371	0.34 [0.29; 0.39]
SEN (59) Child, Diarrhea	125	367	0.34 [0.29; 0.39]
NGA (30) Child, Diarrhea	1858	5416	0.34 [0.33; 0.36]
IND (128) Child, Diarrhea	35	101	0.35 [0.25; 0.45]
IND (94) Child, Diarrhea	12	34	0.35 [0.20; 0.54]
NPL (192) Child, Diarrhea	382	1077	0.35 [0.33; 0.38]
CHN (201) Combination, Gastroenteritis or other etiologies	337	948	0.36 [0.32; 0.39]
KEN (41) Child, Diarrhea	1858	5154	0.36 [0.35; 0.37]
IND (192) Child, Diarrhea	360	974	0.37 [0.34; 0.40]
CHN (146) Combination, Gastroenteritis or other etiologies	171	445	0.38 [0.34; 0.43]
GTM (17) Child, Diarrhea	35	90	0.39 [0.29; 0.50]
IDN (115) Combination, Diarrhea	3140	8074	0.39 [0.38; 0.40]
TZA (114) Child, Diarrhea	35	89	0.39 [0.29; 0.50]
ZAF (73) Child, Diarrhea	13	33	0.39 [0.23; 0.58]
KEN (142) Combination, Severe diarrhea or cholera	4	10	0.40 [0.12; 0.74]
BFA (111) Child, Diarrhea	455	1067	0.43 [0.40; 0.46]
MRT (195) Combination, Diarrhea	62	144	0.43 [0.35; 0.52]
PSE (2) Combination, Diarrhea	96	221	0.43 [0.37; 0.50]
KEN (53) Child, Diarrhea	721	1656	0.44 [0.41; 0.46]
ETH (27) Child, Diarrhea	197	452	0.44 [0.39; 0.48]
COD (154) Combination, Diarrhea	34	76	0.45 [0.33; 0.57]
PSE (2) Combination, Diarrhea	91	200	0.46 [0.38; 0.53]
IND (37) Child, Diarrhea	144	303	0.48 [0.42; 0.53]
CHN (49) Self, Diarrhea	87	183	0.48 [0.40; 0.55]
TZA (192) Child, Diarrhea	297	623	0.48 [0.44; 0.52]
IND (80) Child, Diarrhea	44	92	0.48 [0.37; 0.58]
MOZ (124) Child, Diarrhea	150	313	0.48 [0.42; 0.54]
NGA (19) Child, Diarrhea	142	294	0.48 [0.42; 0.54]
KEN (41) Child, Diarrhea	133	275	0.48 [0.42; 0.54]
UGA (158) Child, Diarrhea	64	130	0.49 [0.40; 0.58]
NGA (126) Child, Diarrhea	9	18	0.50 [0.26; 0.74]
ZAF (130) Child, Diarrhea	4	8	0.50 [0.16; 0.84]
KEN (41) Child, Severe diarrhea or cholera	3314	6524	0.51 [0.50; 0.52]
THA (109) Child, Diarrhea	24	47	0.51 [0.36; 0.66]
CHN (49) Child, Diarrhea	10	19	0.53 [0.29; 0.76]
KEN (113) Child, Diarrhea	210	389	0.54 [0.49; 0.59]
ETH (21) Child, Diarrhea	44	80	0.55 [0.43; 0.66]
ZAF (73) Child, Diarrhea	11	20	0.55 [0.32; 0.77]
MOZ (8) Child, Diarrhea	291	510	0.57 [0.53; 0.61]
BGD (86) Child, Diarrhea	413	723	0.57 [0.53; 0.61]
BGD (192) Child, Diarrhea	985	1670	0.59 [0.57; 0.61]
BGD (4) Child, Severe diarrhea or cholera	22	36	0.61 [0.43; 0.77]
GHA (165) Child, Gastroenteritis or other etiologies	11	18	0.61 [0.36; 0.83]
HTI (50) Child, Severe diarrhea or cholera	141	225	0.63 [0.56; 0.69]
HTI (50) Combination, Severe diarrhea or cholera	1155	1803	0.64 [0.62; 0.66]
HTI (69) Combination, Severe diarrhea or cholera	56	87	0.64 [0.53; 0.74]
ETH (197) Child, Diarrhea	207	321	0.64 [0.59; 0.70]
MWI (107) Child, Diarrhea	128	194	0.66 [0.59; 0.73]
KEN (142) Child, Severe diarrhea or cholera	2	3	0.67 [0.09; 0.99]
IND (48) Child, Diarrhea	113	169	0.67 [0.59; 0.74]
BGD (186) Combination, Severe diarrhea or cholera	3154	4716	0.67 [0.66; 0.68]
KEN (140) Child, Diarrhea	566	833	0.68 [0.65; 0.71]
KEN (202) Child, Diarrhea	72	103	0.70 [0.60; 0.79]
NER (112) Child, Diarrhea	750	1066	0.70 [0.68; 0.73]

BDI (117) Child, Diarrhea	207	294	0.70	[0.65; 0.76]
ETH (150) Child, Diarrhea	431	610	0.71	[0.67; 0.74]
PAK (120) Child, Diarrhea	241	339	0.71	[0.66; 0.76]
CHN (136) Combination, Gastroenteritis or other etiologies	76	104	0.73	[0.63; 0.81]
BGD (186) Combination, Severe diarrhea or cholera	30	41	0.73	[0.57; 0.86]
ETH (133) Child, Diarrhea	172	233	0.74	[0.68; 0.79]
NGA (76) Child, Diarrhea	621	831	0.75	[0.72; 0.78]
CHN (68) Child, Diarrhea	795	1040	0.76	[0.74; 0.79]
ETH (173) Child, Diarrhea	236	297	0.79	[0.74; 0.84]
CHN (45) Child, Diarrhea	6	7	0.86	[0.42; 1.00]
CHN (45) Combination, Diarrhea	25	29	0.86	[0.68; 0.96]
CMR (54) Combination, Severe diarrhea or cholera	85	97	0.88	[0.79; 0.93]
IND (18) Child, Diarrhea	51	57	0.89	[0.78; 0.96]
RWA (87) Child, Severe diarrhea or cholera	155	168	0.92	[0.87; 0.96]
BGD (186) Combination, Severe diarrhea or cholera	1681	1813	0.93	[0.91; 0.94]
PAK (1) Self, Diarrhea	317	338	0.94	[0.91; 0.96]
ETH (77) Child, Diarrhea	219	232	0.94	[0.91; 0.97]
**Random effects model**		**136664**	**0.33**	**[0.28; 0.38]**
**Prediction interval**				**[0.03; 0.87]**

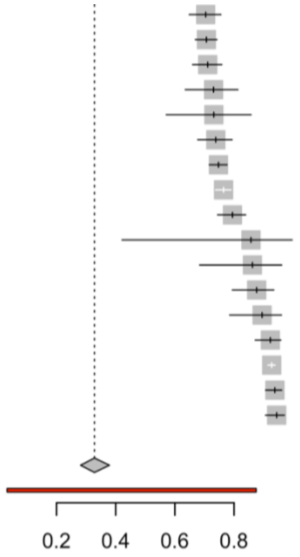

Heterogeneity: I^2 = 99%, τ^2 = 1.8011, p = 0

Figure J. Forest plot of hypothetical care seeking at a hospital or clinic in LMICs

Estimated proportion that would have sought care for a hypothetical episode of diarrheal illness at a hospital or clinic across the 29 observations included in the random-effects meta-analysis.

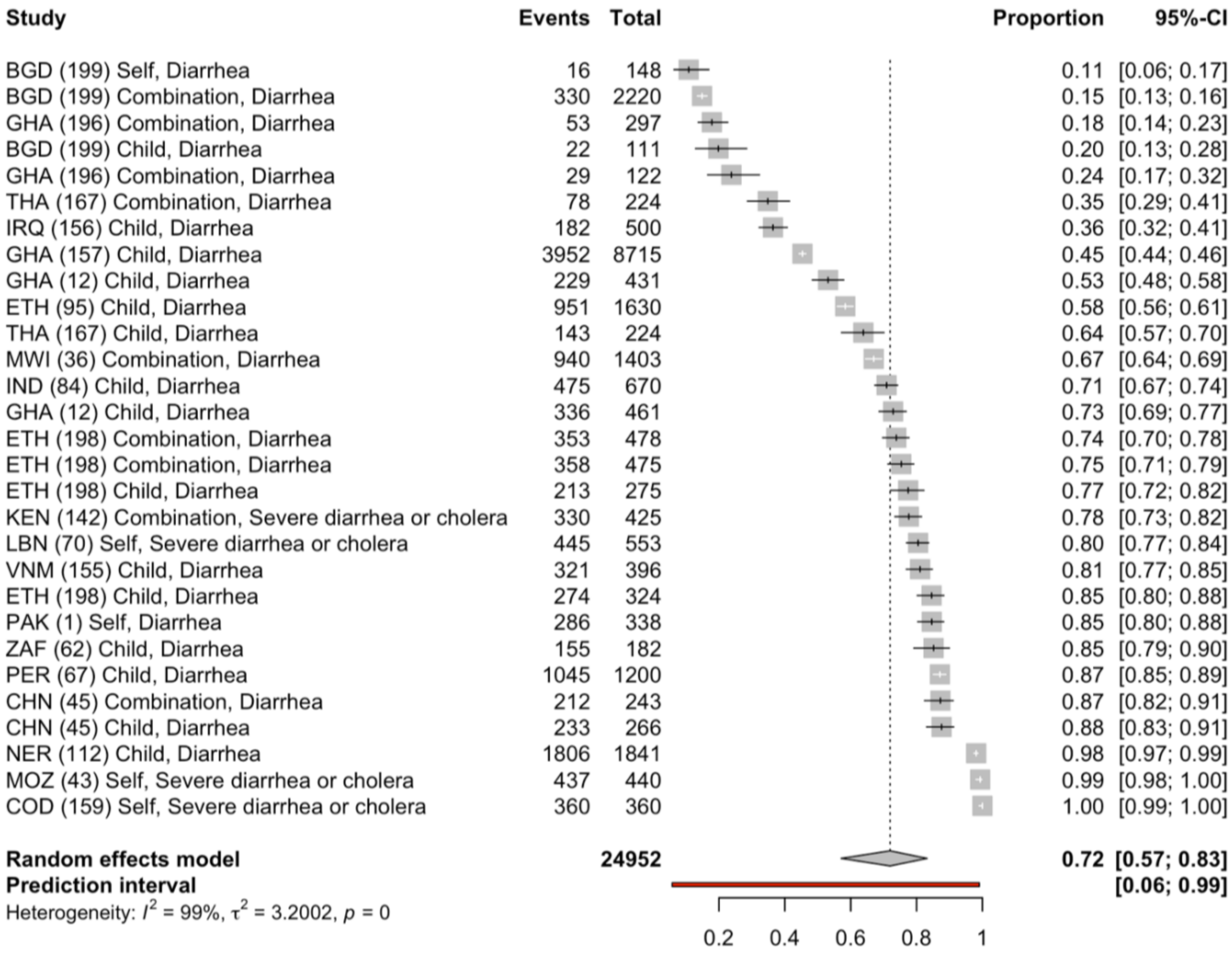

Figure K. Forest plot of hypothetical care seeking at a hospital or clinic in HICs

Estimated proportion that sought care for an actual episode of diarrheal illness at a hospital or clinic across the 24 observations from high-income countries (HICs) included in the random-effects meta-analysis.

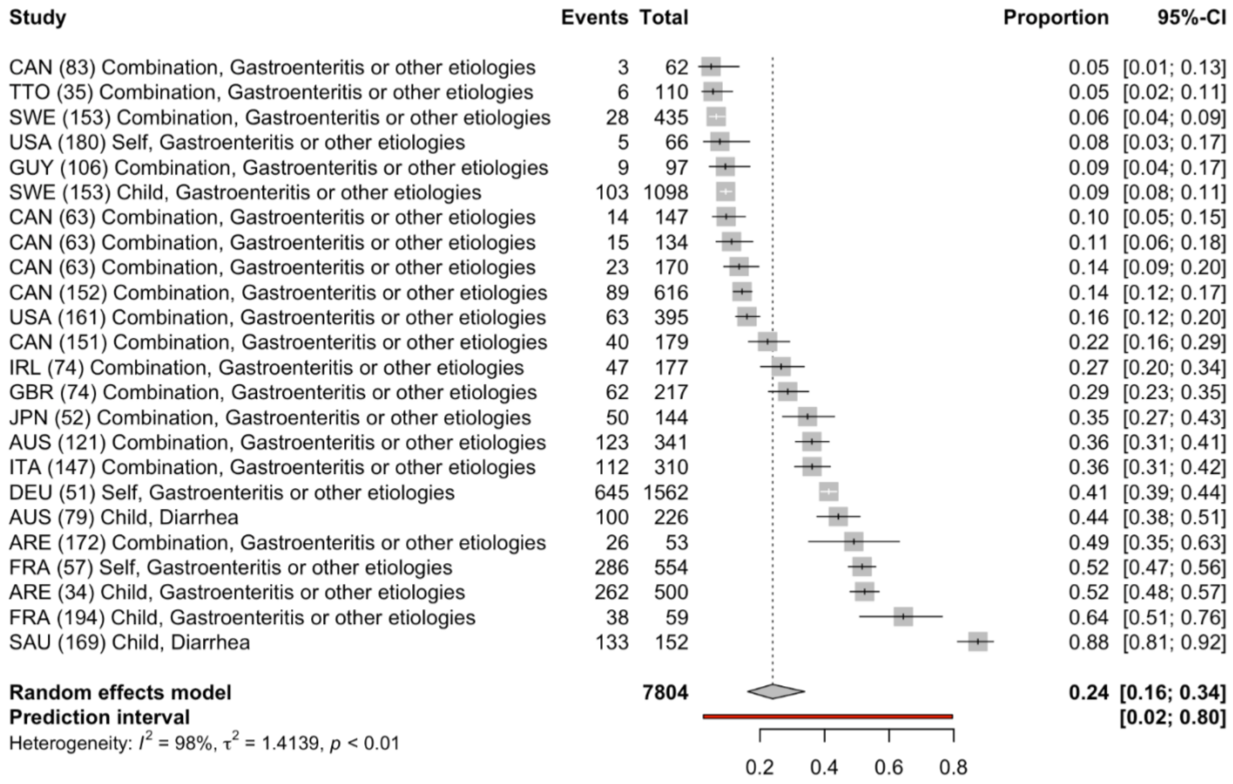

Figure L. Forest plot of care seeking for general diarrhea in LMICs

Estimated proportion that sought care at a hospital or clinic across the 122 in LMICs studies that used a general diarrhea case definition.

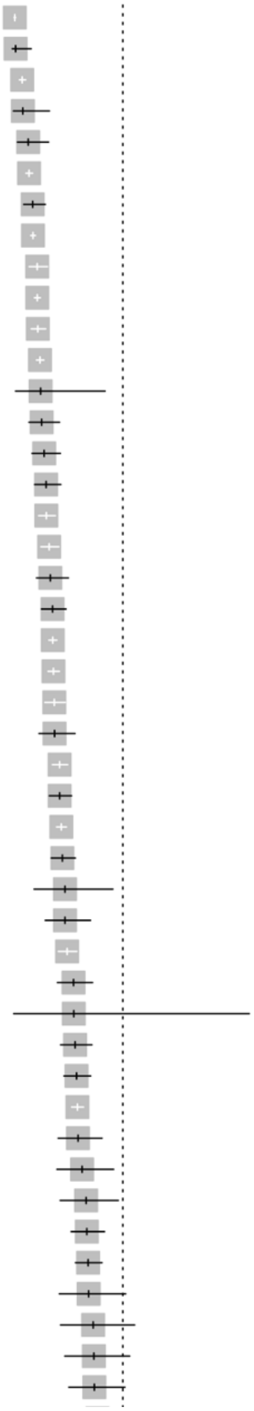

Study	Events	Total	Proportion	95%-CI
IND (125) Child	58	8038	0.01	[0.01; 0.01]
BGD (26) Child	1	106	0.01	[0.00; 0.05]
IND (134) Child	41	1508	0.03	[0.02; 0.04]
IND (145) Combination	2	69	0.03	[0.00; 0.10]
KEN (15) Combination	5	115	0.04	[0.01; 0.10]
IND (85) Child	98	2145	0.05	[0.04; 0.06]
BGD (31) Child	15	270	0.06	[0.03; 0.09]
BGD (28) Combination	291	5156	0.06	[0.05; 0.06]
IND (24) Combination	29	428	0.07	[0.05; 0.10]
BGD (92) Child	275	4049	0.07	[0.06; 0.08]
UGA (178) Child	49	706	0.07	[0.05; 0.09]
IND (100) Child	211	2800	0.08	[0.07; 0.09]
IND (175) Self	2	26	0.08	[0.01; 0.25]
KEN (15) Child	15	188	0.08	[0.05; 0.13]
LBR (101) Child	20	231	0.09	[0.05; 0.13]
PAK (163) Combination	26	283	0.09	[0.06; 0.13]
BGD (116) Child	62	671	0.09	[0.07; 0.12]
PAK (163) Child	63	629	0.10	[0.08; 0.13]
GTM (17) Combination	22	213	0.10	[0.07; 0.15]
GTM (17) Self	40	366	0.11	[0.08; 0.15]
CMR (127) Child	367	3342	0.11	[0.10; 0.12]
UGA (178) Child	185	1657	0.11	[0.10; 0.13]
BRA (108) Combination	61	535	0.11	[0.09; 0.14]
TZA (23) Child	21	183	0.11	[0.07; 0.17]
MWI (36) Child	136	1059	0.13	[0.11; 0.15]
MLI (171) Child	65	506	0.13	[0.10; 0.16]
BGD (129) Child	333	2504	0.13	[0.12; 0.15]
UGA (203) Child	61	449	0.14	[0.11; 0.17]
KEN (123) Combination	7	49	0.14	[0.06; 0.27]
UGA (203) Combination	20	140	0.14	[0.09; 0.21]
KEN (91) Child	121	814	0.15	[0.12; 0.17]
MMR (32) Child	42	253	0.17	[0.12; 0.22]
THA (93) Self	1	6	0.17	[0.00; 0.64]
ZAF (192) Child	55	323	0.17	[0.13; 0.22]
KEN (13) Child	78	448	0.17	[0.14; 0.21]
ZMB (33) Child	392	2220	0.18	[0.16; 0.19]
GTM (17) Self	31	174	0.18	[0.12; 0.24]
GTM (17) Combination	21	111	0.19	[0.12; 0.27]
GHA (12) Child	22	110	0.20	[0.13; 0.29]
UGA (137) Child	65	322	0.20	[0.16; 0.25]
CHN (102) Combination	105	511	0.21	[0.17; 0.24]
ZAF (22) Combination	18	87	0.21	[0.13; 0.31]
GHA (166) Combination	16	73	0.22	[0.13; 0.33]
BGD (25) Child	21	95	0.22	[0.14; 0.32]
MLI (171) Child	28	126	0.22	[0.15; 0.30]

BGD (99) Self	48	208	0.23	[0.18; 0.29]
NIC (9) Child	54	232	0.23	[0.18; 0.29]
BGD (92) Child	762	3258	0.23	[0.22; 0.25]
UGA (46) Child	165	685	0.24	[0.21; 0.27]
KEN (131) Combination	3339	13724	0.24	[0.24; 0.25]
PAK (192) Child	809	3210	0.25	[0.24; 0.27]
GTM (17) Child	44	168	0.26	[0.20; 0.34]
ETH (82) Child	242	904	0.27	[0.24; 0.30]
HTI (141) Combination	14	52	0.27	[0.16; 0.41]
AFG (72) Child	30	110	0.27	[0.19; 0.37]
GHA (12) Child	37	128	0.29	[0.21; 0.38]
MDG (96) Child	96	332	0.29	[0.24; 0.34]
JOR (135) Combination	32	109	0.29	[0.21; 0.39]
THA (167) Combination	11	37	0.30	[0.16; 0.47]
PER (192) Child	632	2102	0.30	[0.28; 0.32]
ETH (88) Child	94	304	0.31	[0.26; 0.36]
ETH (3) Child	36	116	0.31	[0.23; 0.40]
ZMB (89) Child	101	319	0.32	[0.27; 0.37]
BRA (192) Child	58	180	0.32	[0.25; 0.40]
PER (39) Child	14	43	0.33	[0.19; 0.49]
ETH (168) Child	196	596	0.33	[0.29; 0.37]
KEN (131) Child	2981	8817	0.34	[0.33; 0.35]
KEN (113) Child	126	371	0.34	[0.29; 0.39]
SEN (59) Child	125	367	0.34	[0.29; 0.39]
NGA (30) Child	1858	5416	0.34	[0.33; 0.36]
IND (128) Child	35	101	0.35	[0.25; 0.45]
IND (94) Child	12	34	0.35	[0.20; 0.54]
NPL (192) Child	382	1077	0.35	[0.33; 0.38]
KEN (41) Child	1858	5154	0.36	[0.35; 0.37]
IND (192) Child	360	974	0.37	[0.34; 0.40]
GTM (17) Child	35	90	0.39	[0.29; 0.50]
IDN (115) Combination	3140	8074	0.39	[0.38; 0.40]
TZA (114) Child	35	89	0.39	[0.29; 0.50]
ZAF (73) Child	13	33	0.39	[0.23; 0.58]
BFA (111) Child	455	1067	0.43	[0.40; 0.46]
MRT (195) Combination	62	144	0.43	[0.35; 0.52]
PSE (2) Combination	96	221	0.43	[0.37; 0.50]
KEN (53) Child	721	1656	0.44	[0.41; 0.46]
ETH (27) Child	197	452	0.44	[0.39; 0.48]
COD (154) Combination	34	76	0.45	[0.33; 0.57]
PSE (2) Combination	91	200	0.46	[0.38; 0.53]
IND (37) Child	144	303	0.48	[0.42; 0.53]
CHN (49) Self	87	183	0.48	[0.40; 0.55]
TZA (192) Child	297	623	0.48	[0.44; 0.52]
IND (80) Child	44	92	0.48	[0.37; 0.58]
MOZ (124) Child	150	313	0.48	[0.42; 0.54]
NGA (19) Child	142	294	0.48	[0.42; 0.54]
KEN (41) Child	133	275	0.48	[0.42; 0.54]
UGA (158) Child	64	130	0.49	[0.40; 0.58]
NGA (126) Child	9	18	0.50	[0.26; 0.74]
ZAF (130) Child	4	8	0.50	[0.16; 0.84]
THA (109) Child	24	47	0.51	[0.36; 0.66]
CHN (49) Child	10	19	0.53	[0.29; 0.76]
KEN (113) Child	210	389	0.54	[0.49; 0.59]

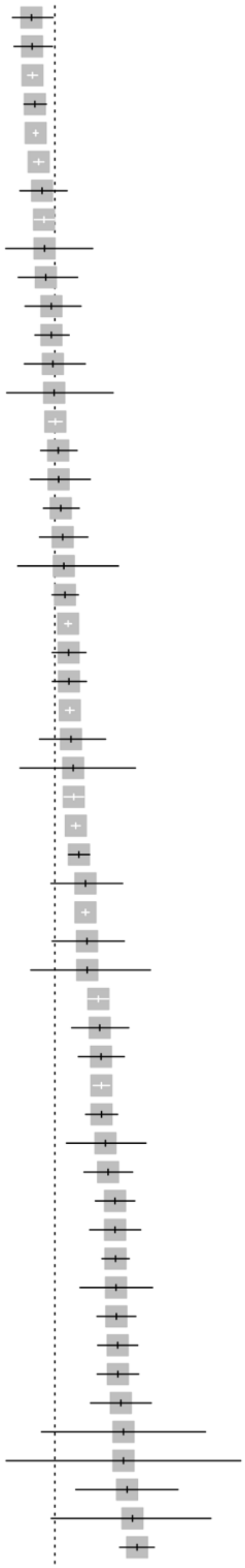

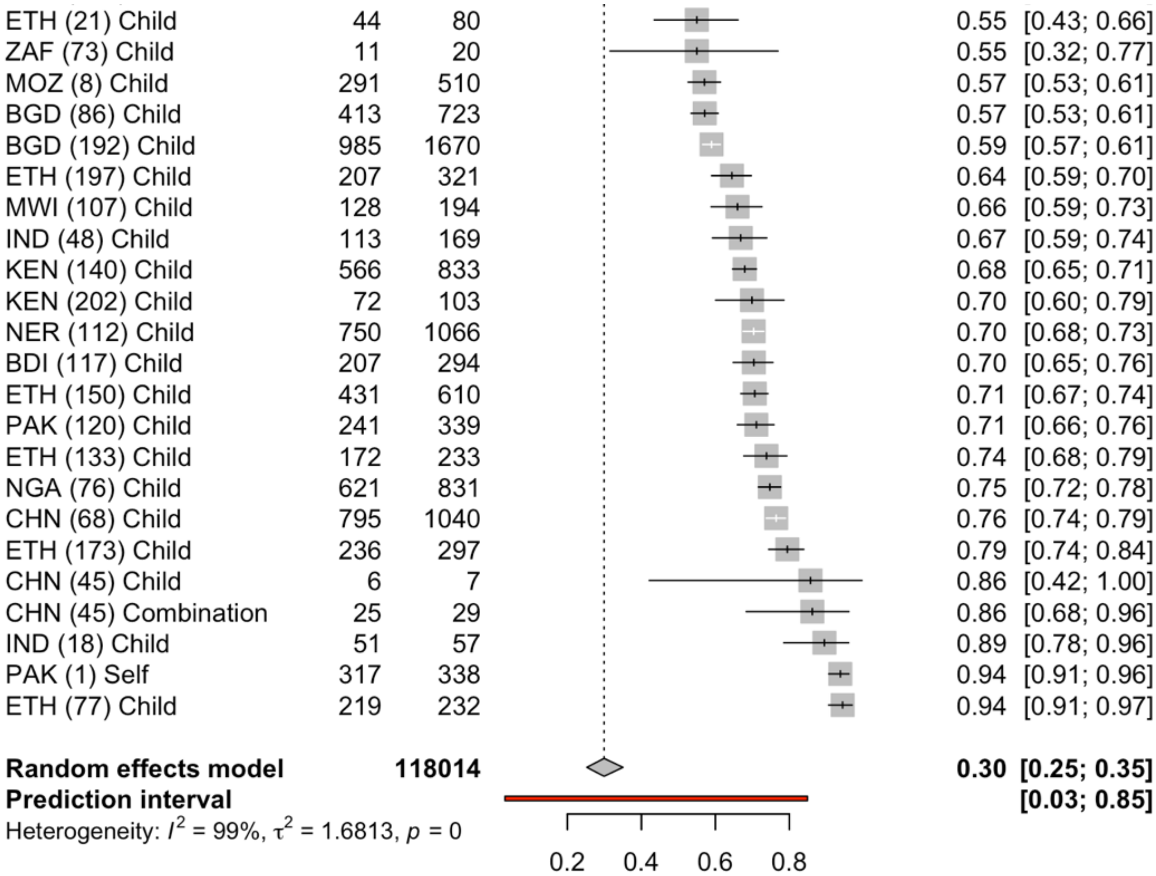
ETH (21) Child 44 80 0.55 [0.43; 0.66]
ZAF (73) Child 11 20 0.55 [0.32; 0.77]
MOZ (8) Child 291 510 0.57 [0.53; 0.61]
BGD (86) Child 413 723 0.57 [0.53; 0.61]
BGD (192) Child 985 1670 0.59 [0.57; 0.61]
ETH (197) Child 207 321 0.64 [0.59; 0.70]
MWI (107) Child 128 194 0.66 [0.59; 0.73]
IND (48) Child 113 169 0.67 [0.59; 0.74]
KEN (140) Child 566 833 0.68 [0.65; 0.71]
KEN (202) Child 72 103 0.70 [0.60; 0.79]
NER (112) Child 750 1066 0.70 [0.68; 0.73]
BDI (117) Child 207 294 0.70 [0.65; 0.76]
ETH (150) Child 431 610 0.71 [0.67; 0.74]
PAK (120) Child 241 339 0.71 [0.66; 0.76]
ETH (133) Child 172 233 0.74 [0.68; 0.79]
NGA (76) Child 621 831 0.75 [0.72; 0.78]
CHN (68) Child 795 1040 0.76 [0.74; 0.79]
ETH (173) Child 236 297 0.79 [0.74; 0.84]
CHN (45) Child 6 7 0.86 [0.42; 1.00]
CHN (45) Combination 25 29 0.86 [0.68; 0.96]
IND (18) Child 51 57 0.89 [0.78; 0.96]
PAK (1) Self 317 338 0.94 [0.91; 0.96]
ETH (77) Child 219 232 0.94 [0.91; 0.97]
Random effects model 118014 0.30 [0.25; 0.35]
Prediction interval [0.03; 0.85]
Heterogeneity: I^2 = 99%, τ^2 = 1.6813, p = 0
0.2 0.4 0.6 0.8

Figure M. Forest plot of care seeking for severe diarrhea or cholera in LMICs

Estimated proportion that sought care at a hospital or clinic across the 15 studies in LMICs that used a severe diarrhea or cholera case definition, including deaths.

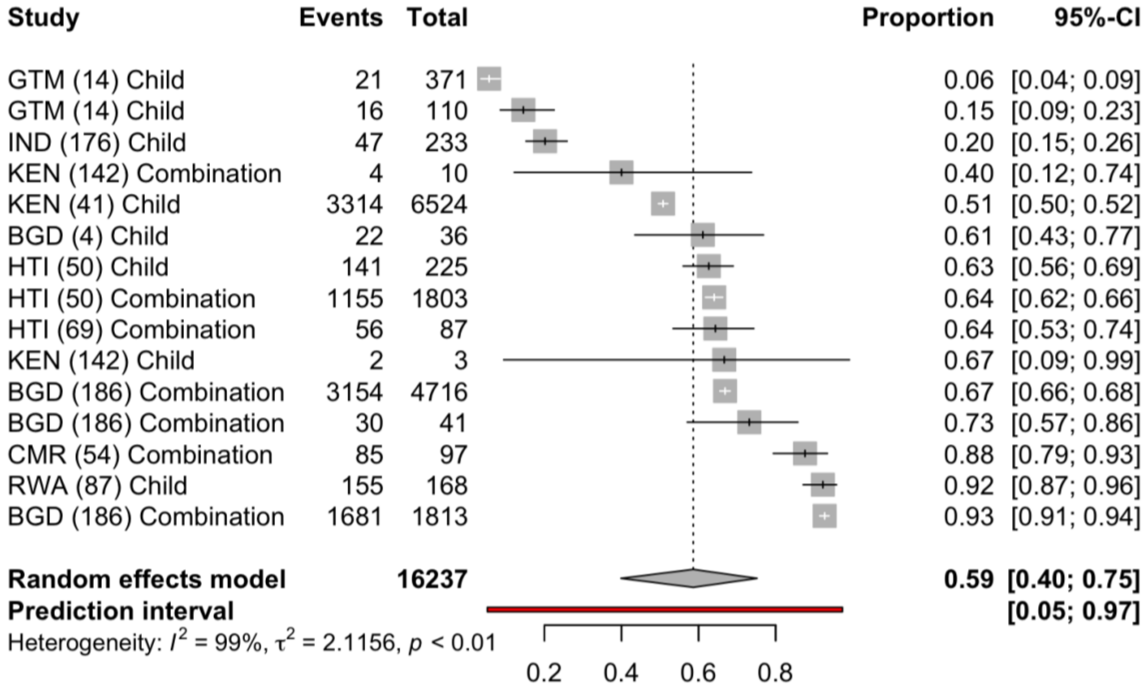

Figure N. Forest plot of care seeking for gastroenteritis or other etiologies in LMICs

Estimated proportion that sought care at a hospital or clinic across the 8 studies in LMICs that used a gastroenteritis or other etiologies case definition.

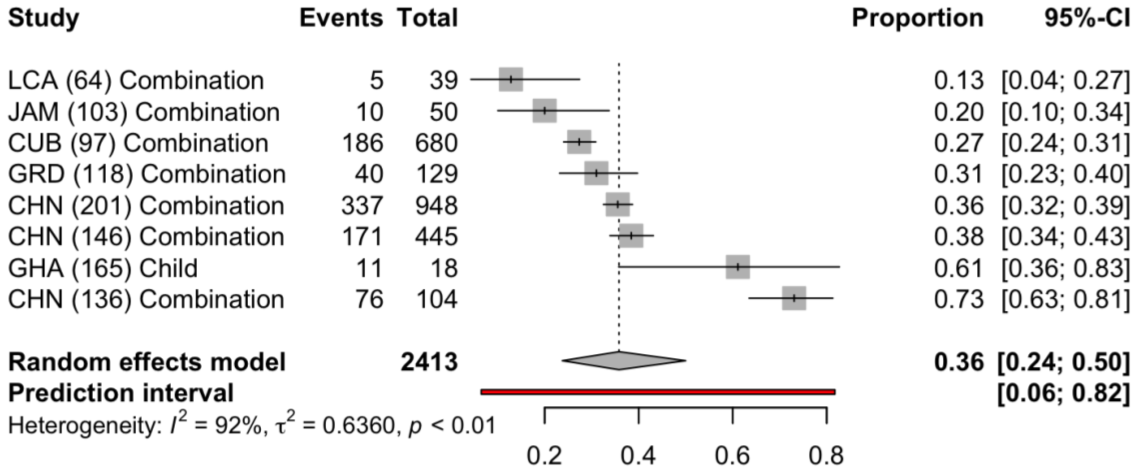

Section C: Supplementary Tables

Table A. Standardized categories for sources of care and examples from studies

Standardized categories of sources of care sought outside the home and examples from included studies of options that fell into each of these categories.

**Source of care**	**Example descriptions from papers**
Any care outside the home	● Any non-specified source outside the home
Hospital or clinic	● Doctor or licensed practitioner<br>● Hospital or clinic<br>● Qualified healthcare provider<br>● Cholera treatment center<br>● Free church-run clinic
Public hospital or clinic	● Public hospital or clinic<br>● Public health center or facility<br>● District hospital<br>● Government clinic<br>● Government healthcare facility
Private hospital or clinic	● Private hospital<br>● Private practitioner<br>● Private care provider
Pharmacy	● Pharmacy or pharmacist<br>● Shop, market, or kiosk<br>● Drug or medicine seller<br>● Chemist
Health post	● Health post<br>● Oral rehydration point<br>● Informal health clinic<br>● Community health center<br>● Dispensary
Community health worker	● Community health worker<br>● Midwife<br>● Village health volunteer<br>● Community distributor
Traditional Healer	● Traditional healer or provider<br>● Spiritual healer<br>● Religious leader<br>● Traditional medicine<br>● Bush doctor<br>● Homeopath
Friends/Family	● Neighbor<br>● Friend<br>● Relative
Other	● Unlicensed provider<br>● Informal help<br>● Village doctor<br>● Non-health facility<br>● Other

Table B. Univariate analyses of factors associated with variation in care seeking

Odds of seeking care for diarrhea at a hospital or clinic in a LMIC for each indicated variable category compared to the reference group, adjusting for no other variables. p-values are shown for the univariate mixed-effects models; n.s. indicates not significant (p > 0.05).

**Variable**	**Category**	**Odds ratio**	**p-value**
Diarrhea case definition	Diarrhea	1 [Reference]	
	Severe diarrhea or cholera	3.26 (1.60 - 6.64)	< 0.01
	Gastroenteritis or other etiologies	1.3 (0.51 - 3.32)	n.s.
Study location type	Non-urban (Rural, Peri-Urban, and IDP or Refugee Camp)	1 [Reference]	
	Urban	1.08 (0.66 - 1.76)	n.s.
	Urban and non-urban	1.12 (0.59 - 2.12)	n.s.
Multiple choices for types of care sought	No	1 [Reference]	
	Yes	1.00 (0.55 - 1.79)	n.s.
During or recently after an outbreak that causes diarrhea	No	1 [Reference]	
	Yes	3.15 (1.38 – 7.18)	< 0.01
Survey recall period	42-365 days or not reported	1 [Reference]	
	2-30 days	0.44 (0.26 - 0.73)	< 0.01
Relationship of diarrhea case to survey respondent	Child (of survey respondent)	1 [Reference]	
	Combination (child, self, or other family member)	0.91 (0.54 - 1.52)	n.s.
	Self (survey respondent)	0.88 (0.30 - 2.57)	n.s.
Timing of care seeking	Care sought at any time	1 [Reference]	
	First source sought	0.46 (0.25 – 0.86)	< 0.05

Table C. Tests for potential confounding with severe diarrhea or cholera case definition

Odds that an observation in a LMIC used severe diarrhea or cholera as the case definition instead of general diarrhea for each category compared to the reference group. p-values are shown for the univariate mixed-effects models; n.s. indicates not significant ($p > 0.05$).

**Variable**	**Category**	**Odds ratio**	**p-value**
Multiple choices for types of care sought	No	1 [Reference]	
	Yes	0.93 (0.45 - 1.91)	n.s.
During or recently after an outbreak that causes diarrhea	No	1 [Reference]	
	Yes	3.52 (1.29 - 9.63)	< 0.05
Survey recall period	42-365 days or not reported	1 [Reference]	
	2-30 days	0.47 (0.24 - 0.92)	< 0.05
Relationship of diarrhea case to survey respondent	Child (of survey respondent)	1 [Reference]	
	Combination (child, self, or other family member)	1.40 (0.72 - 2.73)	n.s.
	Self (survey respondent)	0.84 (0.24 - 2.93)	n.s.
Timing of care seeking	Care sought at any time	1 [Reference]	
	First source sought	1.11 (0.51 - 2.41)	n.s.

Table D. Sub-analyses of associations with outbreak context and recall period

Odds of seeking care at a hospital or clinic in a LMIC when a study describes an outbreak compared to when it does not among 15 studies with severe diarrhea or cholera case definition, and odds of seeking care when a study uses a recall period of 2-30 compared to longer recall periods among the 122 studies that used a general diarrhea case definition. p-values are shown for the univariate mixed-effects models; n.s. indicates not significant ($p > 0.05$).

**Case definition for sub-analysis**	**Variable**	**Category**	**Odds ratio**	**p-value**
Severe diarrhea or cholera (n = 15)	During or recently after an outbreak that causes diarrhea	No	1 [Reference]	
		Yes	1.52 (0.31 - 7.54)	n.s.
Diarrhea (n = 122)	Study recall period	42-365 days or not reported	1 [Reference]	
		2-30 days	0.74 (0.39 - 1.43)	n.s.
