# Supplementary material for: Care-seeking for diarrheal illness: A systematic review and meta-analysis
Source: PLOS Glob Public Health. 2025 Apr 22;5(4):e0004483. doi: 10.1371/journal.pgph.0004483 (PMC12013865; doi:10.1371/journal.pgph.0004483)
Supplement: Attachment — Submitted filename: PGPH_RTR.docx [file pgph.0004483.s006.docx]

**Journal Requirements:**

1. Please provide separate figure files in .tif or .eps format.

For more information about figure files please see our guidelines:
<https://journals.plos.org/globalpublichealth/s/figures> 
<https://journals.plos.org/globalpublichealth/s/figures#loc-file-requirements>

Response: We have uploaded revised figures that follow the above guidelines and have been checked by the PACE tool.

2. Please provide an Author Summary. This should appear in your manuscript between the Abstract (if applicable) and the Introduction, and should be 150–200 words long. The aim should be to make your findings accessible to a wide audience that includes both scientists and non-scientists. Sample summaries can be found on our website under Submission Guidelines:
<https://journals.plos.org/globalpublichealth/s/submission-guidelines#loc-parts-of-a-submission>.

Response: We have added an author summary.

3. As required by our policy on Data Availability, please ensure your manuscript or supplementary information includes the following:

- A numbered table of all studies identified in the literature search, including those that were excluded from the analyses.
- For every excluded study, the table should list the reason(s) for exclusion.
- If any of the included studies are unpublished, include a link (URL) to the primary source or detailed information about how the content can be accessed.
- A table of all data extracted from the primary research sources for the systematic review and/or meta-analysis. The table must include the following information for each study:
- Name of data extractors and date of data extraction
- Confirmation that the study was eligible to be included in the review.
- All data extracted from each study for the reported systematic review and/or meta-analysis that would be needed to replicate your analyses.
- If data or supporting information were obtained from another source (e.g. correspondence with the author of the original research article), please provide the source of data and dates on which the data/information were obtained by your research group.
- If applicable for your analysis, a table showing the completed risk of bias and quality/certainty assessments for each study or outcome.  Please ensure this is provided for each domain or parameter assessed. For example, if you used the Cochrane risk-of-bias tool for randomized trials, provide answers to each of the signalling questions for each study. If you used GRADE to assess certainty of evidence, provide judgements about each of the quality of evidence factor. This should be provided for each outcome.
- An explanation of how missing data were handled.
- This information can be included in the main text, supplementary information, or relevant data repository. Please note that providing these underlying data is a requirement for publication in this journal, and if these data are not provided your manuscript might be rejected.

Response: The initial submission was compliant with all data availability requirements above that were in line with PRISMA guidelines; specific details are included in the S1 Checklist. In addition, in this revision we have added an Excel file (S2 Data) that details all 722 studies included in full-text screening and the reasons for exclusion, where applicable. Since we did not record the date of extraction during the data collection process, we are not able to add that information retroactively to S1 Data.

**Comments to the Author**

**Reviewer #1:** Overall: This is a systematic review and meta-analysis focusing on global care-seeking behaviors for diarrheal illness. The review and data abstraction was methodically sound. However, due to the large volume of data abstracted and analyzed, there are some sections where the data stratifications (i.e., which data are included in a particular analysis) are unclear. In addition, the broad scope of the paper and number of results makes it difficult to draw meaningful conclusions. It would be helpful to narrow down the scope of the paper (e.g., care-seeking behaviors for hospitals and clinics in LMICs) and limit conclusions to those that directly support the research questions.

Response: Thank you for this feedback. We have narrowed down the scope of the paper as suggested by restricting the focus to care seeking behaviors in LMICs. We have also gone through to make sure that we are clear about what data is included in each analysis, as detailed below in response to the specific comments.

Specific comments:
• Lines 186 – 188: Are there any citations to support the statement that private hospitals are rarely part of surveillance systems? This likely varies greatly by country, and if these are truly not generally part of surveillance systems it would underscore a need to capture care-seeking and diarrheal burden in these facilities. If separating these out, it would be worth conducting a sensitivity analysis comparing care-seeking behaviors among those explicitly defined as private hospitals with public hospitals.

Response: We have added references justifying our exclusion of private hospitals from the overall meta-analysis on lines 322-324 in the tracked changes version of the revised manuscript. As shown in Figure 4, care-seeking behavior was generally lower at private hospitals and clinics compared to public or general hospitals and clinics. While factors influencing care-seeking at private versus public facilities may differ, this was not the focus of our study. In response to the original comment about narrowing the scope, we have not elaborated further on this analysis, as our research specifically focuses on care-seeking at hospitals or clinics where individuals are more likely to be captured by surveillance systems.

• Lines 258 – 262: The authors state that they have excluded clinics from this section unless otherwise stated, but most of the section (i.e., lines 272 – 285) seems to include clinics. For clarity, would suggest completely separating these studies out into their own section, or including them and conducting a sensitivity analysis.

Response: Thank you for catching this. We have clarified throughout that studies sampling individuals or caregivers at clinics, rather than in homes or community settings, were excluded from our main analyses. Please see lines 322-326 in the tracked changes version of the manuscript, also copied below.

“*Our primary focus was care-seeking at hospitals or clinics, where people can receive treatment and are most often counted in disease surveillance efforts [22,23], in low- and middle-income countries (LMICs). Since care-seeking behavior among individuals surveyed at a hospital or clinic likely differs from that of individuals surveyed in the broader community, we excluded observations with clinic-based surveillance from all the following the analyses unless otherwise noted.”*

In line with the reviewers’ suggestion, we kept the separate analysis of the clinic-based studies on lines 426-433 in the tracked changes version of the revised manuscript. Given the small sample size, we did not perform a meta-analysis of these studies. We clarified what the study population was for this analysis in the plot titles for S1 Appendix Fig S8, also copied below.

• Lines 413 – 415: If the survey questions/underlying data represent where respondents would seek care, it may not be an appropriate conclusion to state that a certain percentage would not seek care at hospitals/clinics.

Response: This result refers to care seeking for actual (not hypothetical) diarrheal episodes. As suggested by Reviewer 2, we have revised the beginning of the Discussion to read as follows: “Our findings reveal that most people with diarrhea do not seek care at hospitals or clinics. This is important as frequently only individuals attending hospital are counted in typical disease surveillance systems. Furthermore, it may indicate gaps in treatment access.”

• Please describe any potential differences in care-seeking behaviors in papers that included sampling during the COVID-19 pandemic.

Response: This is an interesting idea! However, the review includes only nine observations from studies where data collection overlapped with the COVID-19 pandemic. Given the limited data available to adequately address this question, and in response to the original comment to focus the paper on the main research questions, we have not included an additional analysis.

**Reviewer #2:** This is an excellent piece of research and deserves to be published with only some minor stylistic revisions. The methodological rigor of the review is excellent, with methods described in wonderful detail, and with transparency throughout. The paper addresses an important research question which is clearly outlined, and the authors illustrate the gap their research fills. The only revisions I would recommend are minor linguistic/stylistic edits as follows:

I would argue that the first sentence of the abstract would read better as "Monitoring and treating diarrheal illness often relies...", instead of "Monitoring and treating diarrheal illness rely.." as I feel the subject of the sentence "Monitoring and treating diarrheal illness" is essentially singular here not plural, as it acts as an unified singular concept, grammatically being a compound gerund phrase.

Response: Thank you, we have revised this to “relies”.

Otherwise, I would ensure that "care seeking" is written as "care-seeking" throughout the text. Both versions are currently used, with "care seeking" featuring throughout the piece.

Response: We have corrected this to “care-seeking” throughout.

I feel the first line of the discussion could be stronger. "Here we estimated that most people with diarrhea (on average 67%) do not seek care at hospitals or clinics where they can receive treatment when needed and be counted in typical surveillance systems". Perhaps something like "Our findings reveal that most people with diarrhea do not seek care at hospitals or clinics. This is important as frequently only individuals attending hospital are counted in typical disease surveillance systems. Furthermore, it may indicate gaps in treatment access".

Response: We have revised this sentence as suggested.

I also think lines 193-195 could be improved. It could be worthwhile explicitly stating why the maximum values were taken, given the risk of overestimation. However, I understand this method is simple and consistent. The phrasing itself is quite complicated: "When aggregating data from studies with multiple choice questions where there were multiple different options within one category or source of care (e.g., multiple different hospitals or clinics that could have been selected), we took the maximum value across those options." Something along the lines of the following sentence may be clearer:

"For studies with multiple-choice questions that provided several options within the same category (e.g., public hospitals, private hospitals, or community clinics under 'source of care'), we used the highest percentage reported within the category to represent the overall care-seeking behavior for that category in our analysis."

Response: We have clarified this sentence as suggested, with some revisions for accuracy:

“*For studies with multiple-choice questions that provided several options within the same category (e.g., multiple different public hospitals and/or community clinics under 'source of care'), we used the highest percentage reported within the category to represent the overall care-seeking behavior for that category in our analysis.”*

Lastly, I would revise the final paragraph of the article. The conclusion feels a little weak and I feel could be improved to highlight the importance of your piece and its findings, the urgency to address gaps and how your findings can drive actionable change.

Response: Thank you, and we agree. We have revised the final paragraph, copied below, which we believe strengthens the conclusions.

“*Our findings suggest that care-seeking estimates for broadly defined diarrhea may not reflect true patterns, particularly for severe cases. While we observed trends across age groups, differences in care-seeking between children under five and older individuals were not statistically significant, underscoring the need for more granular data. DHS data can provide a reasonable approximation of care-seeking for non-severe, non-etiology-specific diarrhea, but assumptions about age-related care-seeking should be setting-specific, as adult behaviors varied across contexts. More accurate estimates of care-seeking behaviors can help translate imperfect observations of disease in the population into more reliable assessments of true burden, ultimately supporting the design of better interventions to ensure timely access to appropriate diarrhea treatment across different populations.”*

Overall though, this is a great piece of work and deserves to be published.

Response: Thank you for the kind words.
